# Selective Sensing of Gas Mixture via a Temperature Modulation Approach: New Strategy for Potentiometric Gas Sensor Obtaining Satisfactory Discriminating Features

**DOI:** 10.3390/s17030573

**Published:** 2017-03-12

**Authors:** Fu-an Li, Han Jin, Jinxia Wang, Jie Zou, Jiawen Jian

**Affiliations:** 1 Gas Sensors & Sensing Technology Lab., Faculty of Electrical Engineering and Computer Science, Ningbo University, Ningbo 315211, China; fu.anli@163.com (F.L.); jinhan@nbu.edu.cn (H.J.); 2 School of Information Engineering, Huangshan University, Huangshan 245021, China; 3 School of Electronic and Information Engineering, Ningbo University of Technology, Ningbo 315211, China; wangjx@nbut.edu.cn

**Keywords:** Temperature modulation, PCA, discriminating features, In_2_O_3_, ZnO

## Abstract

A new strategy to discriminate four types of hazardous gases is proposed in this research. Through modulating the operating temperature and the processing response signal with a pattern recognition algorithm, a gas sensor consisting of a single sensing electrode, i.e., ZnO/In_2_O_3_ composite, is designed to differentiate NO_2_, NH_3_, C_3_H_6_, CO within the level of 50–400 ppm. Results indicate that with adding 15 wt.% ZnO to In_2_O_3_, the sensor fabricated at 900 °C shows optimal sensing characteristics in detecting all the studied gases. Moreover, with the aid of the principle component analysis (PCA) algorithm, the sensor operating in the temperature modulation mode demonstrates acceptable discrimination features. The satisfactory discrimination features disclose the future that it is possible to differentiate gas mixture efficiently through operating a single electrode sensor at temperature modulation mode.

## 1. Introduction

Currently, air pollutants such as nitrogen oxides (NO, NO_2_), carbon monoxide (CO) and hydrocarbons (HCs) derived from vehicles and industrial factories are a concerning issue, due to their effect of generating photochemical smog and airpocalypse [1]. Hence, strict regulations to reduce the emissions of these air pollutants have been implemented worldwide [2,3]. For the purpose of reducing the air pollutants efficiently and/or monitoring the air quality, high-performance gas sensors have to be developed and are considered crucial for improving the performance of combustion controllers and three-way catalysts [4,5].

To date, numerous types of gas sensors, which include but are not limited to the sensing principles of chemiresistive, amperometric, potentiometric and impedancimetric, have been reported [6,7,8,9,10]. Among them, yttria-stabilized zirconia (YSZ)-based electrochemical sensors consisting of a In_2_O_3_ or ZnO sensing electrode (SE) exhibit great potential to sense NO_x_, CO, HCs and NH_3_ because of their reliable performance in harsh conditions [11,12,13,14,15,16,17,18,19,20,21,22,23]. However, overcoming their poor selectivity is still a challenging issue for these sensors. To promote selectivity, tuning the microstructure of the SEs, e.g., the morphology, thickness, and crystal phase, is usually considered, although this approach relies on sophisticated materials synthesis techniques [24,25]. Meanwhile, the strategy to design the sensor array and combine it with specific data processing algorithms also shows promising results in differentiating gas mixtures [6,26,27]. Some examples of data processing algorithms include but are not limited to Fisher discriminant analysis, hierarchical clustering analysis, artificial neural networks, simple fuzzy logic and principal component analysis (PCA) [27,28,29,30,31]. PCA in multivariate statistical analysis is a method of compressing the dimension of vector data by extracting principal typical components from sample data set. It is a custom program, proposed for analyzing sensor output data [6,26,27,28,29]. Nevertheless, complex preparation techniques remarkably raise the cost of sensor arrays, thereby restraining the manufacturing of such devices on a large scale.

Herein, we report a novel strategy to discriminate gas mixtures through modulating the operating temperature. A sensor comprised of a single SE is designed and its sensing characteristics are further investigated to verify the applicability of using the proposed discrimination method in future applications.

## 2. Materials and Methods

### 2.1. Sample Preparation and Fabrication of the Sensor

Commercially available 8YSZ powder (8 % mol Y_2_O_3_ added zirconia, Tosoh, Japan) were used as electrolyte substrate by tape casting. The slurry was prepared by a two-step ball milling procedure. In the first step, the ceramic powder was homogeneously dispersant in a roller ball mill for 24 h using zirconia balls, acrylic resin, xylene, and butyl acetate as milling medium, dispersant, and solvents, respectively. The polyvinyl butyral (PVB) was added as binder and polyethylene glycol (PEG) was used as plasticizer, followed by roller ball mill for another 6 h. The formula of slurry is showed in Table 1. Before tape casting, the slurries were vacuum pumped for 10 min to remove air. The 8YSZ film was casted on Mylar substrate at the blade height of 300 μm. After drying, the thickness of the green 8YSZ tape was maintained at 125 μm. In order to fabricate the thick electrolyte supported layer, six sheets of 8YSZ green tapes were laminated. The 8YSZ green layer was calcined at 1400 °C for 4 h in air to form the 8YSZ ceramic substrate. Finally, the Length, width and thickness were 16, 9 and 0.6 mm, respectively.

**Figure 1:**
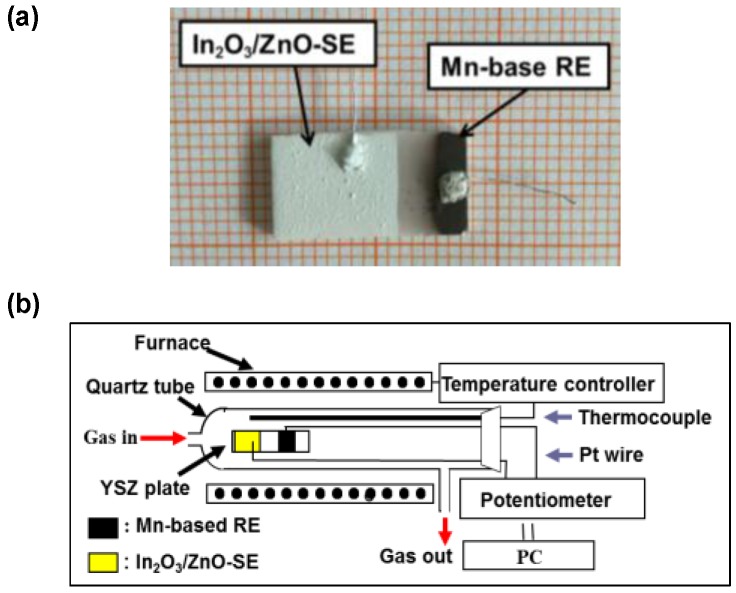
(**a**) Photograph of the fabricated sensor comprised of In_2_O_3_/ZnO composite SE and Mn-based RE; (**b**) schematic view of the gas sensing characteristics evaluation apparatus.

To fabricate the sensor device, commercially available ZnO and In_2_O_3_ (Sinopharm, Shanghai, China) powers were used to prepare the sensing electrodes. ZnO and In_2_O_3_ powders were mixed with the weight ratio of 0, 5, 10, 15, 20, 30 and 100 wt.%, respectively. Afterwards, these powder mixtures were mixed with α-terpineol and screen-printed on the surface of the 8YSZ ceramic plate. These semi-manufactured samples were calcined at 800, 900, 1000 and 1100 °C for 2 h in air, respectively to form the SE. Finally, MnO_2_ paste were screen-printed and calcined in the same way to form the Mn-based reference electrodes (RE). The photographic image of the fabricated sensor is shown in Figure 1a.

### 2.2. Characterization of Sensing Characteristics

The gas sensing characteristics of these sensors were measured in a conventional gas flow apparatus equipped with an electric furnace in the temperature range of 450–700 °C. Both SE and RE are simultaneously exposed to the base gas (5 vol.% O_2_ in a balance of N_2_) or sample gas (NO_2_, CO, C_3_H_6_ and NH_3_ at 50–400 ppm), respectively. The voltages between sensing electrode and reference electrode was measured by a multifunction data acquisition card (HP 34970A, Agilent, Santa Clara, CA, USA). The potential difference *ΔV* was subtracted *V*_base gas_ from *V*_sample gas_, where the *V*_sample gas_ represents the sensing response of the sensor toward sample gas and the *V*_base gas_ represents the sensing response of the sensor to base gas. Figure 1b demonstrates the schematic view of the gas sensing characteristics evaluation apparatus. PCA algorithm was written in-house via MATLAB® R2009a ver. 7.8.0 software (MathWorks© Inc., Natick, MA, USA).

## 3. Results and Discussion

In order to obtain high sensitivity and a desirable discriminatory capability through a simplified sensor configuration, namely a sensor composed of a single SE, the following sensing strategy is proposed: ZnO/In_2_O_3_ composites in different ratios were synthesized and utilized as the SEs to enhance the sensing magnitude. Then, the performance and response patterns to various examined gases for the sensor were evaluated and recorded in the temperature range of 450–700 °C. Finally, the obtained response patterns to the sample gases were processed with the PCA algorithm to investigate the differentiation efficiency. It was expected that due to the distinct difference in the gas-phase catalytic and electrocatalytic activity of the SE to each target gas, response patterns to these four gases would vary with the change of the operating temperature. Since the PCA algorithm is a powerful discriminant analysis tool, particularly when dealing with response patterns with distinctive differences, these four gases were expected to be identified even if the examination was implemented through a sensor consisting of a single SE.

**Figure 2:**
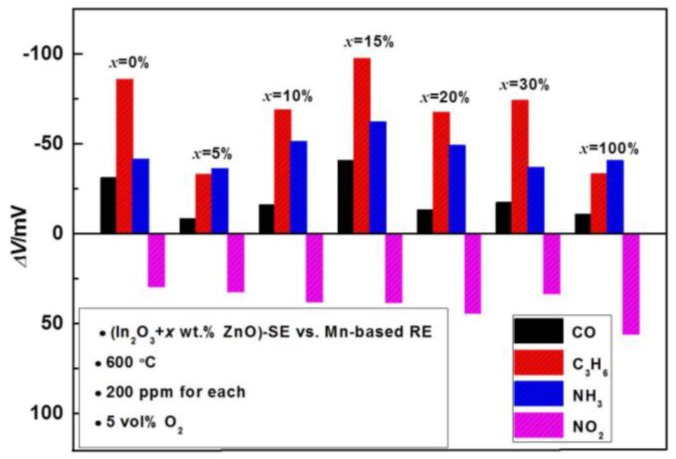
Comparison of the sensing characteristics for the sensors comprised of various composite SEs (calcined at 1000 °C for 2 h) to 200 ppm sample gases, operated at 600 °C.

Initially, the sensitivity and response magnitude of the sensor were optimized. Figure 2 shows the response signal (*ΔV*) to 200 ppm sample gases of the sensors using various composite SEs. It can be concluded that the sensing behavior of the sensors operated at 600 °C is remarkably tuned by the amount of ZnO. For instance, after incorporating 5 wt.% ZnO with In_2_O_3_, the response signal to CO, C_3_H_6_ and NH_3_ suddenly dropped, whereas the addition of 5 wt.% ZnO had a minor effect on the response value of NO_2_. Afterwards, with continuously increasing the ZnO amount, the response signal of the sensors to CO, C_3_H_6_ and NH_3_ increased gradually and reached the maximum value at 15 wt.% ZnO. Then, the response value to these three target gases started to decrease if the ratio of ZnO in the composite was further increased. Note that the sensing performance of the sensor to NO_2_ was relatively less affected by incorporating ZnO when compared with its behavior with other three gases. Hence, in view of the highest sensing signal being observed for most of the examined gases by the addition of 15 wt.% ZnO, the sensor comprised of (In_2_O_3_ + 15 wt.% ZnO) composite SE was used for the following examination.

**Figure 3:**
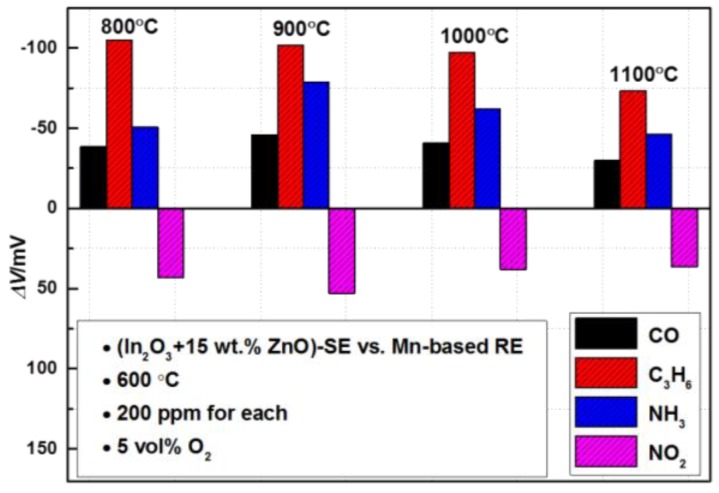
Influence of the calcination temperature (800–1100 °C, with intervals of 100 °C) on the sensing behavior of the sensor comprised of (In_2_O_3_ + 15 wt.% ZnO)-SE, operated at 600 °C.

**Figure 4:**
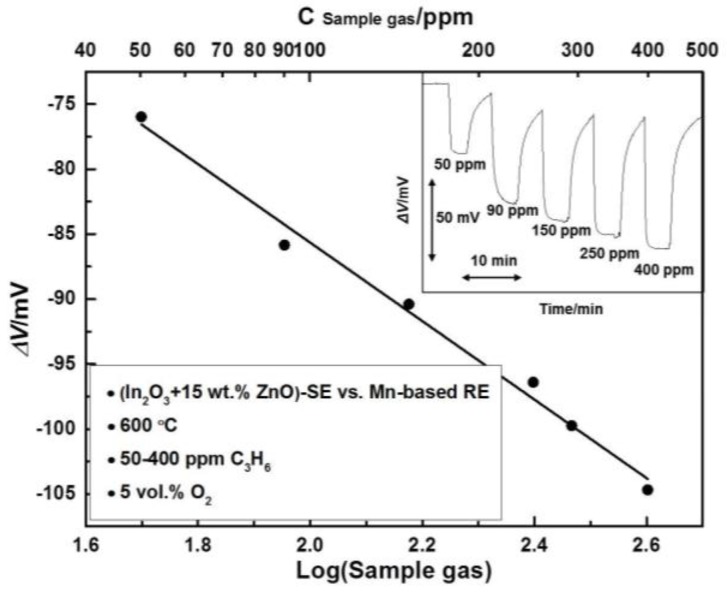
Dependence of the sensing signal (*ΔV*) on the logarithm of C_3_H_6_ concentrations; Insert: response transitions to C_3_H_6_ in the concentration range of 50–400 ppm.

Then, the influence of the calcination temperature was investigated to further enhance the sensing magnitude. After being calcined at temperatures in the range of 800–1100 °C (with intervals of 100 °C), the cross-sensitivity of the sensors with a SE containing 15 wt.% ZnO was examined at 600 °C and is shown in Figure 3. Interestingly, the response magnitude to all the examined gases reached the maximum value when the sensor was fabricated at 900 °C. The *ΔV* measured at 600 °C for 200 ppm CO, NH_3_, C_3_H_6_ and NO_2_ was about −40.64 mV, −62.02 mV, −97.4 mV and 30.08 mV, respectively. Consequently, the optimal calcination temperature for the sensor was selected as 900 °C. Regarding the tuning mechanism of the ZnO and the internal connection between the calcination temperature and the sensing behavior, it is probably due to the gas-phase catalytic and electrocatalytic activity of the composite electrode being closely dependent on its composition and microstructure. This will be thoroughly studied in the near future and will not be discussed in this work, since the main focus of this research is to confirm the feasibility of the strategy in identifying gas mixtures with a single SE. Afterwards, the dependence of the response value on the gas concentration was also examined. For brevity, we list the sensing behavior to C_3_H_6_ herein as the representative example. As shown in Figure 4, the response value of the sensor valid linearly with the logarithm of the C_3_H_6_ concentration; sensitivity of the sensor was roughly estimated as −30.21 mV/Dec. Furthermore, it can be seen from the inserted illustration in Figure 4 that the sensor showed a quick response rate, with a 90% response time to 200 ppm C_3_H_6_ of about 17 s. However, the sensor gave a relatively low recovery rate even when operated at the temperature of 600 °C, e.g., the 90% recovery time to 200 ppm C_3_H_6_ was about 34 s, which needs further improvement in the future.

**Figure 5:**
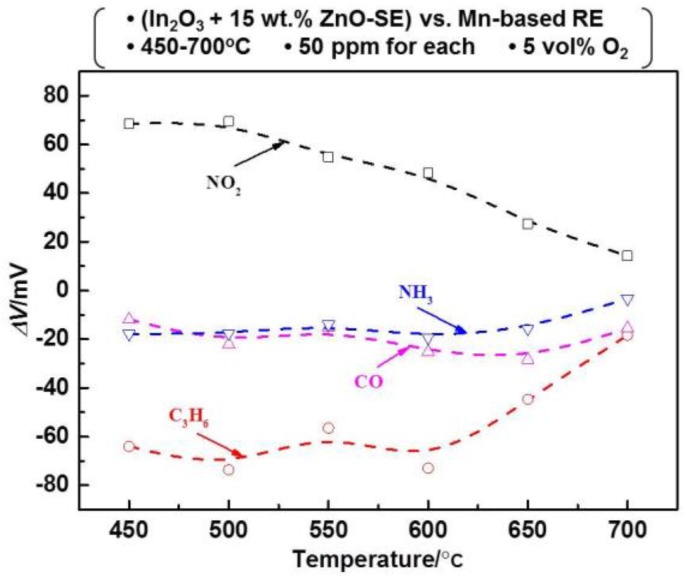
Response patterns of the sensor to the examined gases (50 ppm) at various operating temperatures.

**Figure 6:**
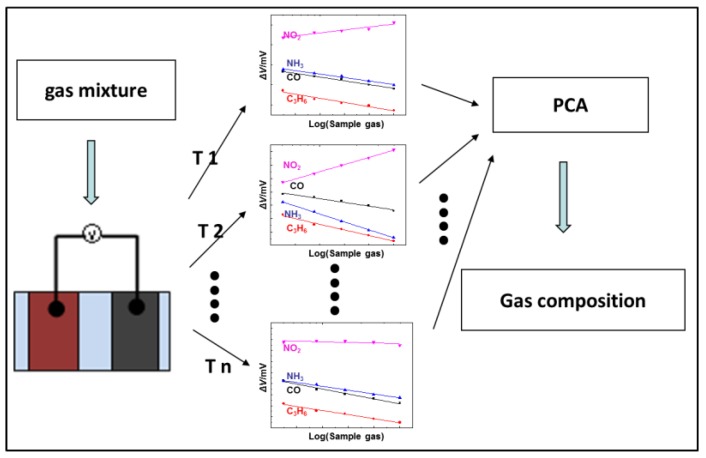
Flowchart of processing the response pattern that was obtained from the temperature modulation mode with PCA software in detail.

Theoretically, high discrimination efficiency can be obtained through operating the sensor in the temperature modulation mode, if distinct differences in the response patterns are observed. Thus, the response patterns to the 50 ppm sample gas were recorded within the operational temperatures of 450–700 °C for the sensor using the (In_2_O_3_ + 15 wt.% ZnO) SE. It can be concluded from the results summarized in Figure 5 that the sensor demonstrated apparent differences in the response patterns to each target gas within the examined temperature range. With the increasing operational temperature, the response value of NO_2_ decreased, whereas the other three sample gases gave a more complex variation in their sensing behavior. Based on these results, it is reasonable to believe that desirable discriminating features will be obtained if processing these response patterns with the PCA algorithm. Consequently, a 20 × 6 (row × column) feature vector (shown in Table 2) was created and input into the PCA software. Each row represents a single measurement at a specific gas concentration within all the examined temperature ranges and each column represents a feature calculated for that particular measurement at a specific temperature. All data obtained from the sensor operated at six different temperatures is equivalent to that given by six different sensors that were operated at the same operational temperature. As a result, a virtual sensor array composed of six sensors was established and applied for sensing these four gases at the level of 50–400 ppm. A flowchart that summarizes the procedure is shown in Figure 6.

**Figure 7:**
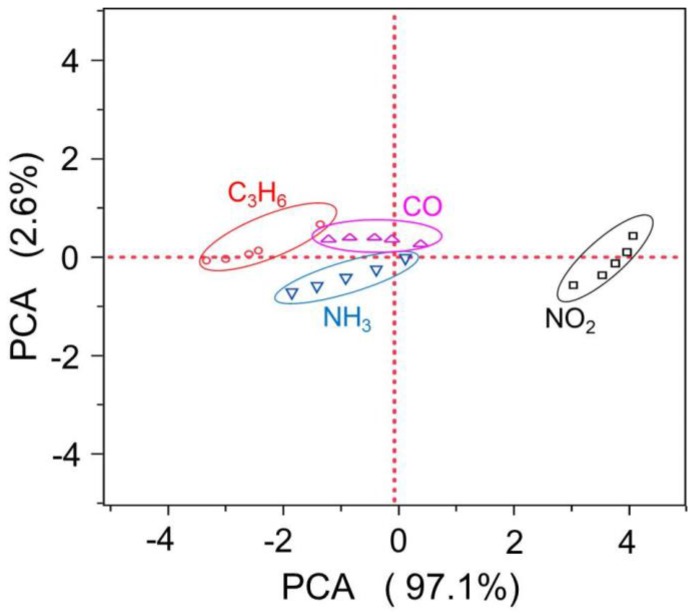
PCA schemes of the data set for the sensor utilizing (In_2_O_3_ + 15 wt.% ZnO)-SE, operated in the temperature modulation mode.

The PCA transformation for all response patterns is shown in Figure 7, with the same colored symbol corresponding to each target gas. The spatial distance between each target gas represents the discrimination capability of the sensor. The overlap of these symbols shown in the PCA scheme implies low differentiating characteristics of the sensor when sensing these symbols representing gas. In contrast, a large spatial distance between these symbols suggests a desirable discrimination feature detected by the sensor array. The results demonstrated in the PCA scheme suggest that the sensor exhibits the best discrimination capability to NO_2_ and acceptable differentiating features to CO, NH_3_ and C_3_H_6_. This is in line with the sensing characteristics shown in Figure 5. As shown in Figure 5, the sensor gave relatively similar response patterns to CO, NH_3_ and C_3_H_6_ within the examined temperature range, whereas distinctive sensing behavior was observed for NO_2_, thereby leading to the satisfactory differentiating feature for NO_2_ and less discriminating capability for CO, NH_3_ and C_3_H_6_. Note that the preliminary success of applying the temperature modulation approach together with the PCA algorithm in discriminating gas mixtures gives a potential method to replace the complex sensor array with a simplified sensor configuration. In particular, it is reasonable to expect that the discrimination features will be further improved if the SE exhibits distinctive response patterns to all the target gases, implying more efforts are needed to develop an appropriate SE in future research.

## 4. Conclusions

Through fabricating a composite SE and operating it in the temperature modulation mode, the sensor consisting of the single SE exhibited an acceptable performance in sensing four types of hazardous gases. With the addition of 15 wt.% ZnO to In_2_O_3_ and being calcined at 900 °C for 2 h, the sensor gave its maximum sensing magnitude to all the examined gases. Moreover, with the aid of the PCA algorithm, acceptable differentiating features were observed through the temperature modulation approach. Such promising results indicate a novel strategy to sense gas mixtures with a simplified potentiometric sensor configuration.

## Figures and Tables

**Table 1:** The formula of electrolyte substrate slurry.

Mill Media		Formula (g)					
Mat.	Initial wt.	acrylic resin	Butyl acetate	Xylene	8YSZ	PVB	PEG
ZrO_2_	800 g	16	25.4	25.4	200	20	10

**Table 2:** The 20 × 6 (row × column) feature vectors being created for PCA analysis.

Sample Gas	Concentration/ppm	Response at Various Operating Temperatures/mV					
			Operating Temperature				
		450 °C	500 °C	550 °C	600 °C	650 °C	700 °C
CO	50	−17.86	−17.58	−13.98	−19.32	−15.64	−3.36
	90	−28.80	−29.52	−30.65	−32.47	−25.07	−8.19
	150	−35.55	−38.18	−38.89	−42.59	−29.41	−13.59
	250	−49.86	−48.76	−47.51	−49.79	−37.41	−20.62
	400	−59.84	−57.19	−55.35	−60.04	−44.59	−28.60
C_3_H_6_	50	−64.00	−73.63	−56.55	−72.98	−44.70	−18.36
	90	−85.79	−90.31	−69.14	−88.84	−81.54	−49.13
	150	−95.33	−87.33	−74.55	−88.39	−85.39	−56.04
	250	−101.95	−92.90	−83.55	−98.40	−94.02	−65.11
	400	−113.98	−106.38	−90.32	−97.68	−101.02	−73.50
NH_3_	50	−11.85	−22.04	−15.27	−25.23	−28.34	−15.47
	90	−20.89	−31.12	−21.69	−32.46	−46.30	−29.93
	150	−27.35	−37.71	−31.58	−50.86	−59.13	−44.26
	250	−40.60	−47.75	−39.50	−57.74	−72.44	−57.34
	400	−50.66	−59.53	−45.50	−65.48	−84.14	−68.49
NO_2_	50	68.49	69.45	54.76	48.34	27.39	14.27
	90	81.07	80.73	57.42	58.15	41.94	27.01
	150	84.90	86.86	56.96	60.37	49.97	39.47
	250	89.57	88.81	54.94	61.28	56.48	50.57
	400	105.53	73.11	49.51	56.35	61.79	63.01
		Sensor 1	Sensor 2	Sensor 3	Sensor 4	Sensor 5	Sensor 6
			Virtual Sensor Array				

## Abbreviations

The following abbreviations are used in this manuscript:

YSZ	Yttria-Stabilized Zirconia
SE	Sensing Electrode
RE	Reference Electrode
PCA	principle component analysis
